# Case study of verrucous carcinoma of the hand: rare location

**DOI:** 10.11604/pamj.2019.33.93.18063

**Published:** 2019-05-28

**Authors:** Mouad Guenbdar, Jamal Louaste

**Affiliations:** 1 Department of Orthopaedics and Traumatology, Military Hospital Moulay Ismail, BP 50000 Meknes, Morocco

**Keywords:** Verrucous, carcinoma, hand

## Image in medicine

Verrucous carcinoma is rare well-differentiated squamous cell carcinoma with very rare metastatic potentiel. Pappilomatosis carcinoides cutis (PPC) is the rarest form of verrucous carcinoma. The risk factors for PCC include chronic inflammation or irritation. It can have multiple locations such as the cheeks, nose, ears, hands, thighs etc. We present a patient with large, cauliflower-shaped mass of the fourth finger. A 60-year-old male presented with painful, cauliflower-shaped mass of 9cm*5cm of the fourth finger of the left hand. The tumor surface was exophytic, irregular, crossed by deep grooves. Disease history is 12 years, the mass was gradually growing. After the histopathological examination of skin biopsy, we have diagnosed pappilomatosis cutis carcinoides. There was no palpable lymphadenopathy. Routine blood investigations were with normal limits. Simple radiographs showed bony erosions. Hand magnetic resonance imaging was performed, an invasion into the bone and tendon was observed. Chest abdominal pelvic CT scan were normal. We performed a partial amputation of the fourth ray with safety margins (5mm) and a full-thickness skin graft. The histopathology of excisional mass border confirmed the tumor was removed entirely. He had no tumor recurrence or metastatic disease during follow-up for 4 years.

**Figure 1:**
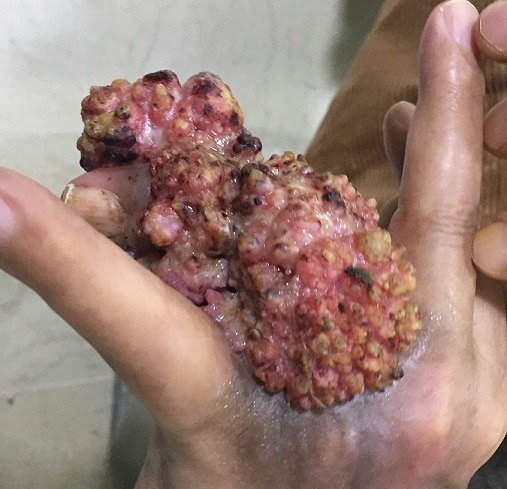
Clinical appearance of verrucous carcinoma of the fourth finger; vegetant mass, cauliflower-shaped with irregular surface

